# Dynamic Signal priority of the self-driving bus at an isolated intersection considering private vehicles

**DOI:** 10.1038/s41598-023-44864-3

**Published:** 2023-10-14

**Authors:** Hui Li, Shuxin Li, Xu Zhang, Pei Tong, Yahui Guo

**Affiliations:** 1 School of Civil Engineering, Henan University of Technology, Zhengzhou, 450001 China; 2 School of Traffic Engineering, Huanghe Jiaotong University, Jiaozuo, 454950 China; 3 School of Civil Engineering, Zhengzhou University, Zhengzhou, 450001 China

**Keywords:** Civil engineering, Environmental impact

## Abstract

The transit signal priority leads to the delay of private vehicles in the priority and non-priority phases. To address this problem, a bi-level programming model is proposed based on the dynamic cycle and arrival rate of private vehicles under connected environment. The upper model is built by a delay triangle, with the maximum delay reduction of private vehicles between the decreased delay and increased delay in the experimental period. The lower model is constructed based on the Stackelberg model of game theory, and the objective is to obtain the dynamic cycle. A genetic algorithm (GA) is implemented to solve the proposed model. Based on SUMO, a case study of a self-driving bus in the city of Zhengzhou is conducted to demonstrate the effectiveness of the proposed model. The results from GA and SUMO are consistent, which verifies the effectiveness of the proposed model. The delay of the private vehicles with dynamic signal priority declines by 21.32% on average compared to that without priority. Compared with active signal priority, it declines by 22.63% on average. The proposed method is compared with the method proposed by other papers, and the delay per private vehicle is small. The effectiveness of the proposed method is further illustrated. The proposed methodology is helpful for improving the operation efficiency of intersections with minimum delay.

## Introduction

With the development of the economy in China, the urbanisation rate is increasing. An increasing number of people choose to work and live in cities, which poses massive challenges to urban transportation. Problems arise, such as traffic jams. However, public transport is an effective way to clear traffic jams. Various countries have built advanced public transportation systems to provide better services to the public^1^. Transit signal priority (TSP) at a signalised intersection is one of the priority strategies. The benefit of TSP is greater than that of the bus lane^2^. The control strategies of TSP are mainly divided into passive priority and active priority strategies^3^. Passive priority is used to implement TSP regardless of whether there is a bus arriving at the intersection, but the signal timings are predetermined to provide priority to buses. The control strategies of passive priority are as follows: adjustment of cycle length, phase splitting, area-wide signal timing plan and metering-vehicles. However, the major disadvantage is that it is not realistic in real-time traffic conditions. Active priority overcomes the limitation of passive priority, and the detectors are placed upstream of the intersection. Then, signal timings are adjusted to grant priority. The conventional bus priority methods used in active priority are red truncation or early green, green extension, phase rotation, phase insertion, phase skipping and green reallocation^4^. Nevertheless, active priority takes less consideration of vehicles and cannot reasonably balance the operating benefits of buses and vehicles. Dynamic (adaptive) priority^5^ is proposed to minimise negative impacts on vehicles, and this priority system consists of three important components: continuous detection, communication links and a signal control algorithm. Therefore, the research based on dynamic (adaptive) signal priority is implemented to reduce the delay of private vehicles by obtaining road information in this paper.

**Figure 1:**
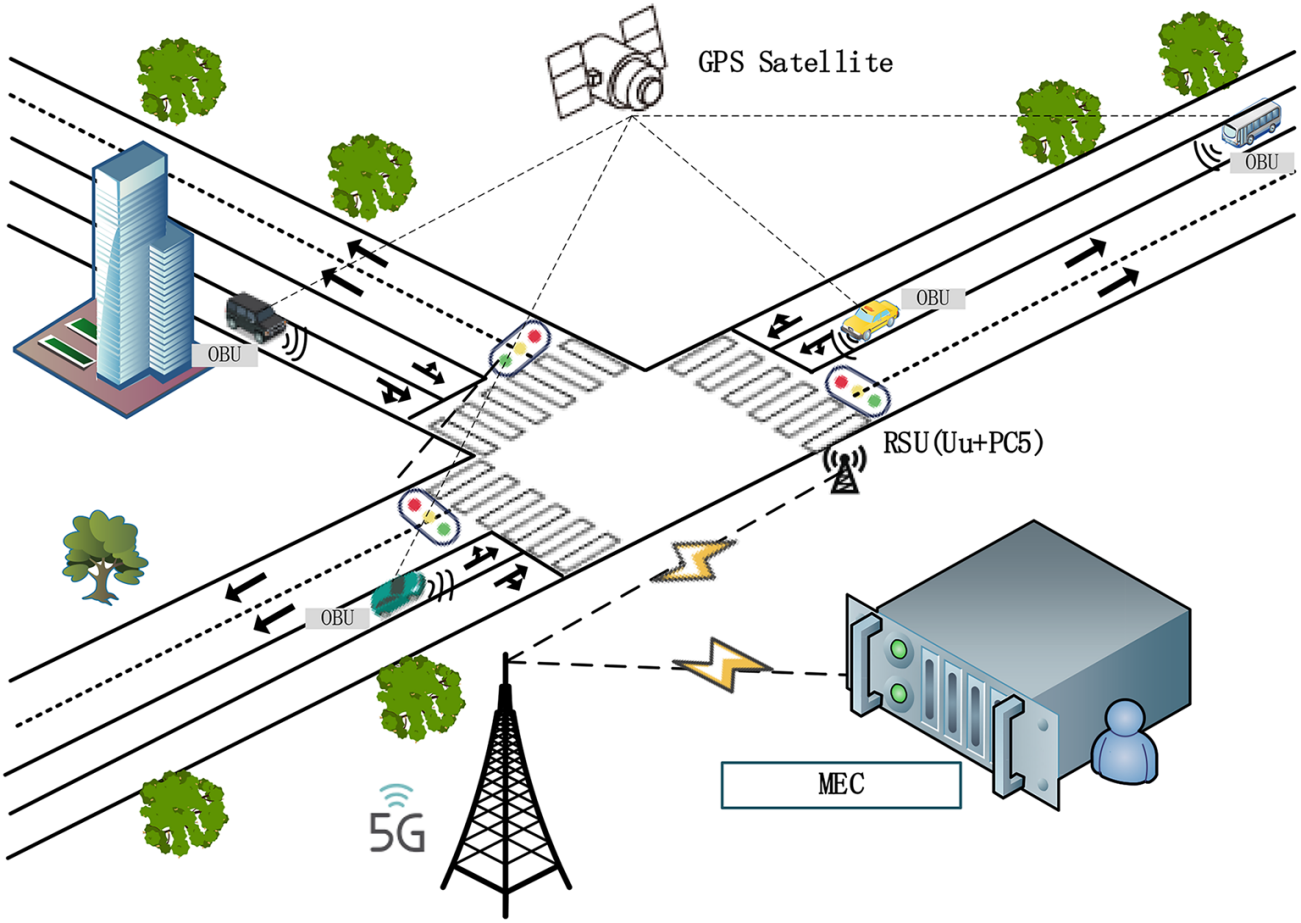
Vehicle information architectural diagram at an intersection^10^.

Vehicle-to-everything (V2X) technology has promoted the development of TSP, and it also provides a theoretical basis for the proposed model in this paper. Based on these technologies, buses can receive more real-time traffic data^6–8^, including the traffic status of other vehicles, traffic signal light data, infrastructure data, etc. When the self-driving bus passes the intersection, the roadside unit (RSU) detects the self-driving bus and private vehicle by connecting with the on-board unit (OBU)^9^. Then, the data of the self-driving bus and private vehicle will be sent to the mobile edge computing (MEC) server, which is connected to the intelligent transportation system of the smart island by a 5G network. Then, the decision information is sent by the MEC server to the RSU. The RSU determines whether to perform the signal control strategy according to whether there is a self-driving bus entering the intersection, and the instructions will be sent to the annunciator. The specific structure is shown in Fig. 1.

TSP has been applied to self-driving buses; however, more attention has been focused on self-driving buses, and less attention has been given to the negative impact of TSP, such as private vehicle delays in the non-priority phase. In some cases, the TSP gain will be offset by the loss from private vehicle delay in the non-priority phase, and even the loss will be greater. Therefore, buses and private vehicles should be considered at the same time. Because V2X technology can exchange information, this paper proposes a model to minimize the delay of private vehicles under the connected environment.

### Literature review

TSP has been established to improve transit operation efficiency; however, it causes negative impacts in the non-priority phase. Taking the TSP into account, while the queue length decreases in priority phase, it increases in non-priority phase^11^. It also will lead to increased delays in non-priority phase^12^. Balancing the benefits of the TSP in the priority phase with the negative effects in the non-priority phase is a critical problem for optimising the effect of the TSP. Girijan et al.^13^ developed the thresholds based on reducing the total person delay, including the other vehicles' delay and the bus delay. The evaluation results indicate a 16.7% to 42.8% reduction in total person intersection delay due to the implemented bus priority. Thodi et al.^14^ constructed the objective function with the minimizing total person delay (including bus delay and other vehicles’ delay). Yu et al.^15^ redefined a mathematical program to minimize total passenger delay, including total car passenger delay and total bus passenger delay. To sum up, the objective function performance is an integrated index, which is a weighted sum of all types of delays.

For the indexes considered in model, Thodi et al.^14^ considered the bus occupancy to other vehicles’ average passenger occupancy ratio and bus-arrival time to the traffic queuing time ratio. Yu et al.^15^ considered uncertain bus arrival times. Wu et al.^16^ considered bus stop locations and bus dwell time durations. Zeng et al.^17^ considered the bus travel time and the time spent at bus stops to find appropriate signal timing. Cvijovic et al.^18^ applied the time that a transit vehicle needs to reach the stop line, the number of passengers on board and the lateness that the transit vehicle experiences to obtain the signal timing. It can be seen from above studies that the bus-oriented traffic indexes are adopted in model.

However, the development of Connected Vehicle (CV) technologies offers the possibility of better balancing the interests of buses and private vehicles^19^. Song et al.^20^ established the model based on minimizing the total person delay under a connected vehicle environment, including the delay of private vehicle and the delay of bus, and considered bus travel time. Hu et al.^21^ reallocated green time based on total person delay, including the delay of vehicle and the delay of bus, considered bus travel time. Feng et al.^22^ presented a real-time adaptive signal phase allocation algorithm using connected vehicle data, and minimisation of total vehicle delay was considered. Yang et al.^23^ provided a TSP algorithm in a connected vehicle environment, considering bus stops and bus schedule.

To address the negative impact in the non-priority phase of TSP, existing researchers have mainly built models to find the optimal signal timing for reducing the delay in the non-priority phase. An integrated index is proposed, which is a weighted sum of all types of delays, such as total person (passage) delay and total vehicle delay, including bus delay and private vehicle delay (in priority and non-priority phases), and the weight of the buses and private vehicles is determined subjectively. The bus-oriented factors are considered, such as, bus stop location, bus arrival rate, bus travel time, bus departure time, and bus dwell time. Vehicles in the non-priority phase are not involved. Therefore, from the perspective of private vehicles in the non-priority phase, this paper presents a bi-level programming model under a connected vehicle environment to determine the dynamic signal timing. The objective function is the maximum delay reduction of private vehicles between the decreased delay and increased delay in the experimental period.

### Objectives and contributions

The objective of this study is to propose a bi-level programming model with the maximum delay reduction of private vehicles between decreased delay and increased delay by compensating for the non-priority phase.

The contributions of the proposed method in this paper are as follows:

1. From the perspective of private vehicles, this paper proposes a bi-level programming model to balance the operating benefits of self-driving buses and private vehicles.
2. A Stackelberg game theory model is developed to obtain the dynamic cycle by combining it with the traffic characteristic of dynamic signal priority.
3. A case study of a self-driving bus in the city of Zhengzhou is used to illustrate the effectiveness of the proposed methodology.

## Model

### Problem description

The existing studies on TSP are mainly focused on the total delays of intersections, including bus delays and private vehicle delays^13–15,20–22^. However, the model is basically built from the perspective of buses^14–18,21,23^, and the bus-oriented indexes are considered. It cannot reasonably balance and coordinate the operating benefits of buses and private vehicles. Therefore, from a more comprehensive perspective, this paper proposes a bi-level programming model with the maximum delay reduction of private vehicles between decreased delay and increased delay in the experimental period. The subject is a self-driving bus, and technologies for the detection of self-driving buses and changes in the signal phase are necessary; therefore, a connected vehicle environment is needed. Due to the above factors, the following assumptions are formulated:

1. It is assumed that buses and private vehicles in the road are in the Level 4 (Highly Autonomous) and equipped with Cooperative Adaptive Cruise Control (CACC). Therefore, bus and private vehicle data, such as speed, location, bus and private vehicle length and signal timing, can be obtained.
2. The delay triangle is used to calculate private vehicle delay, in which the arrival rate and saturation flow rate of the vehicle are very important parameters. To facilitate the calculation of the delay, the arrival rate and the saturation flow rate of the vehicle are assumed to be linear.

### Bi-level programming model

**Figure 2:**
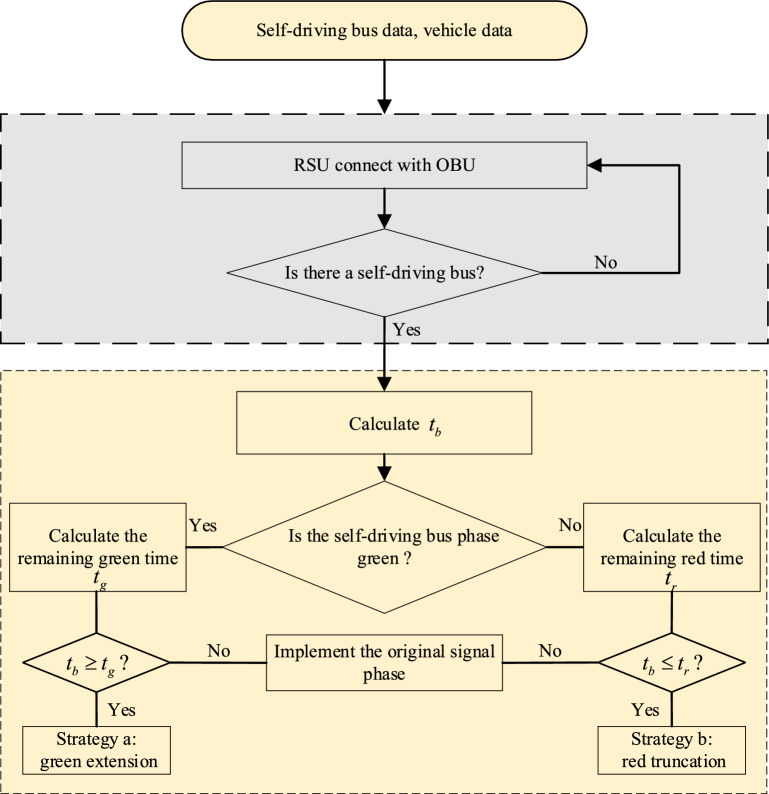
Flow chart of the self-driving bus signal priority control strategy.

Self-driving bus data and private vehicle data can be obtained by connecting the RSU and OBU. When a self-driving bus is detected, the travel time $t_{b}$ of the self-driving bus from the location of the detector to the stop line and the remaining signal time are calculated. The self-driving bus passes the stop line, and the signal is green. The remaining green time $t_{g}$ is calculated. When $t_{b}$ is less than $t_{g}$, the self-driving bus can pass through the intersection according to the original signal phase. When $t_{b}$ is greater than $t_{g}$, the self-driving bus cannot pass through the intersection during the original signal phase. Then, strategy a is implemented, that is, a dynamic cycle is inserted. Green extensions in the priority phase and non-priority phase are carried out to ensure the priority of self-driving buses and mitigate the delay of private vehicles. The signal is red when the self-driving bus arrives at the stop line, and the remaining red time $t_{r}$ is calculated. When $t_{b}$ is greater than $t_{r}$, the self-driving bus can pass through the intersection according to the original signal phase. When $t_{b}$ is less than $t_{r}$, the self-driving bus cannot pass through the intersection by the original signal phase. Then, strategy b is implemented, that is, a dynamic cycle is inserted. Red truncation in the priority phase and non-priority phase is applied to ensure the priority of self-driving buses and mitigate the delay to private vehicles. The flow chart is shown in Fig. 2.

**Figure 3:**
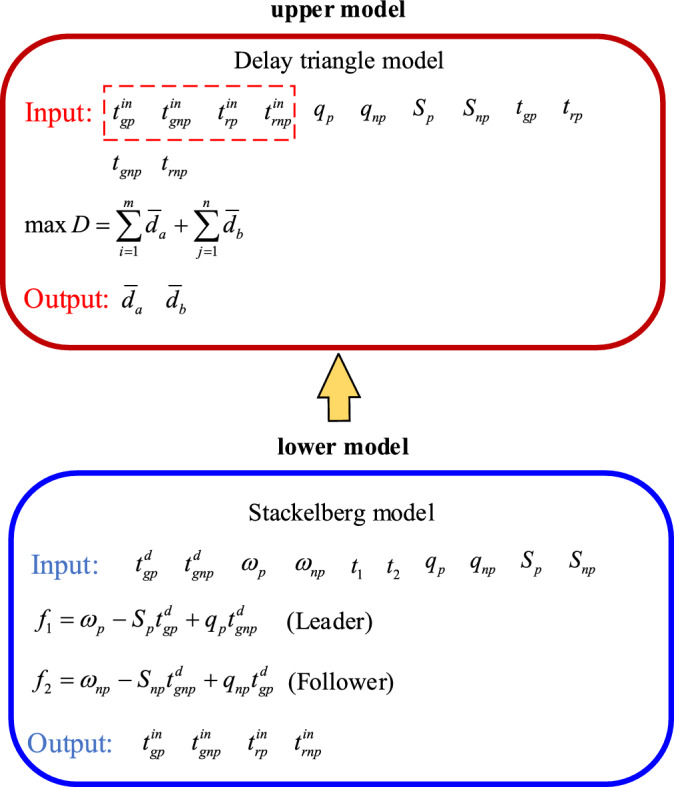
The logical relationship between the upper model and the lower model.

The logical relationship between the upper model and the lower model is shown in Fig. 3. In the figure, the variables $t_{gp}^{in}$ and $t_{gnp}^{in}$ represent the green extension time in priority and non-priority phase, respectively. $t_{rp}^{in}$ and $t_{rnp}^{in}$ are the red truncation time in priority and non-priority phase respectively. The upper model is the delay model, and the lower model is the dynamic phase model. These variables are computed by the lower model and then provided to the upper model where delays can be calculated.

### Upper model

The priority of the self-driving bus is absolute priority, so the delay of the self-driving bus is not considered. In the priority phase, the delay of private vehicles will be decreased due to the priority of self-driving buses. However, the delay of private vehicles in the non-priority phase will increase. The objective function maximises the delay reduction $D$ of private vehicles between a decreased delay and an increased delay under strategies a and b in the experimental period.

1 $$\max D = \sum\limits_{i = 1}^{m} {\overline{d}_{a} } + \sum\limits_{j = 1}^{n} {\overline{d}_{b} }$$

where $\overline{d}_{a}$-Delay reduction of private vehicles between decreased delay and increased delay under strategy a; $\overline{d}_{b}$-Delay reduction of private vehicles between decreased delay and increased delay under strategy b; $m$-The number of dynamic cycles under strategy a during the experimental period; $n$-The number of dynamic cycles under strategy b during the experimental period.

**Figure 4:**
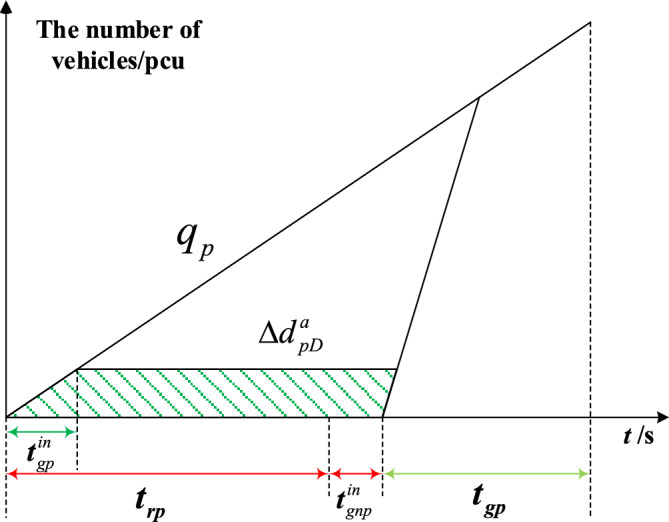
Decreased delay of the priority phase due to green extension in the priority phase under strategy a.

**Figure 5:**
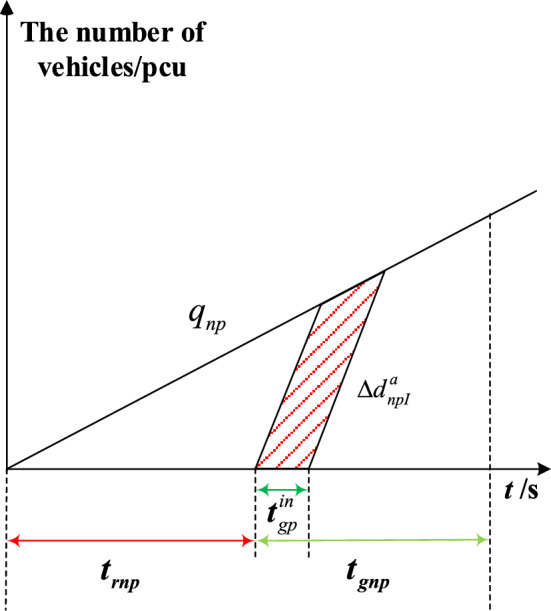
Increased delay of the non-priority phase due to green extension in the priority phase under strategy a.

**Figure 6:**
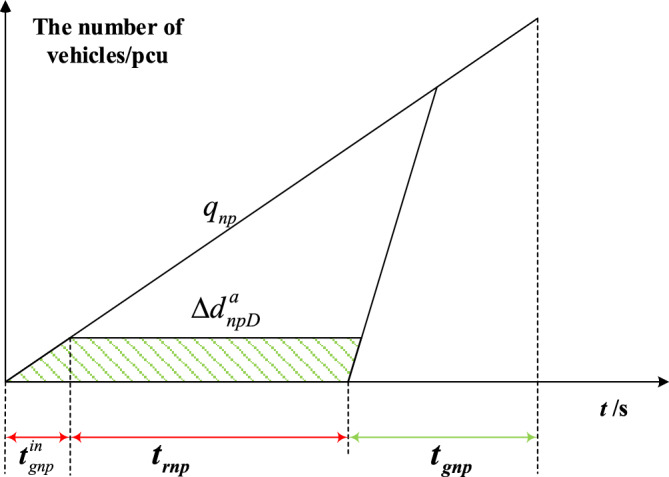
Decreased delay of the non-priority phase due to green extension in the non-priority phase under strategy a.

**Figure 7:**
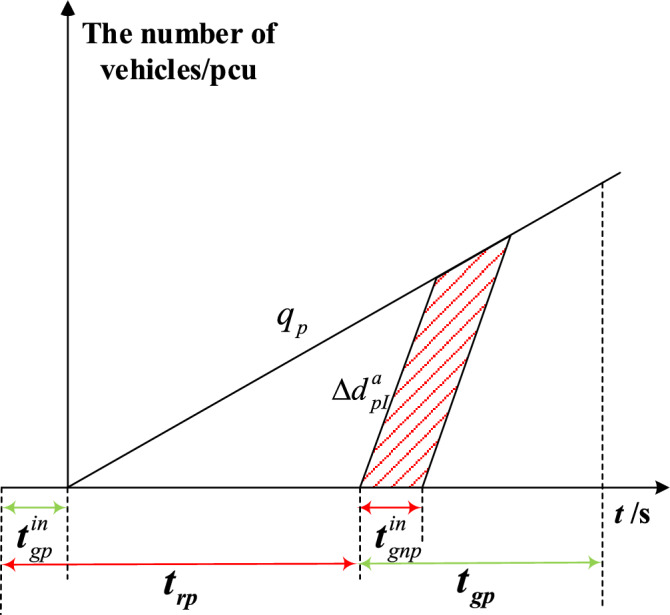
Increased delay of the priority phase due to the green extension in the non-priority phase under strategy a.

The delay triangle^24^ is used to calculate the delay of private vehicles in the priority phase and non-priority phase under strategies a and b. Figures 4, 5, 6 and 7 represent the delay reduction of private vehicles between decreased delay and increased delay when the self-driving bus cannot pass through the intersection in the green phase. Figures 4 and 5 show the delay of private vehicles in the priority phase and non-priority phase due to the green extension in the priority phase. Figures 6 and 7 show the delay of private vehicles in the priority phase and non-priority phase due to the green extension in the non-priority phase.

Figure 4 shows the decreased delay of the priority phase due to the green extension in the priority phase under strategy a. The vertical axis is the number of vehicles before the stop line, and the unit is passenger car unit(pcu). The horizontal axis means the time of traffic signal, and the unit is s. The area surrounded by the arrival curve, saturation release curve and horizontal axis is the total delay of private vehicles in the priority phase. The shaded area is the decreased delay in the priority phase after green extension implementation. Equation (2) calculates the decreased delay of the private vehicles in priority phase due to green extension implemented in priority phase under strategy a.

2 $$\Delta d_{pD}^{a} = \frac{{t_{gp}^{in} q_{p} \left[ {2\left( {t_{rp} + t_{gnp}^{in} } \right) + {{q_{p} \left( {t_{gp}^{in} } \right)} \mathord{\left/ {\vphantom {{q_{p} \left( {t_{gp}^{in} } \right)} {S_{p} - t_{gp}^{in} }}} \right. \kern-0pt} {S_{p} - t_{gp}^{in} }}} \right]}}{2}$$

where $\Delta d_{pD}^{a}$-Decreased delay of the priority phase due to green extension in the priority phase under strategy a; $t_{gp}^{in}$-Green extension time in the priority phase to ensure that the self-driving bus passes through the intersection without stopping; $q_{p}$-Arrival rate of private vehicles in the priority phase; $t_{gp}$-Green time of the priority phase in the original signal phase; $t_{rp}$-Red time of priority phase in original signal phase; $t_{gnp}^{in}$-Green extension time in the non-priority phase to mitigate the increased delay in the non-priority phase; $S_{p}$-Saturation flow rate in the priority phase.

The increased delay of the non-priority phase due to green extension in the priority phase under strategy a is shown in Fig. 5. The shaded area is the increased delay in the non-priority phase after green extension implementation. Equation (3) calculates the increased delay of private vehicles in non-priority phases when the green extension is implemented in priority phases under strategy a.

3 $$\Delta d_{npI}^{a} = \frac{{t_{gp}^{in} q_{np} \left( {2t_{rnp} + t_{gp}^{in} } \right)}}{{2\left( {1 - {{q_{np} } \mathord{\left/ {\vphantom {{q_{np} } {S_{np} }}} \right. \kern-0pt} {S_{np} }}} \right)}}$$

where $\Delta d_{npI}^{a}$-Increased delay of the non-priority phase due to green extension in the priority phase under strategy a; $q_{np}$-Arrival rate of private vehicles in the non-priority phase; $t_{rnp}$-Red time of the non-priority phase in the original signal phase; $S_{np}$-Saturation flow rate in the non-priority phase.

The decreased delay of the non-priority phase due to the green extension in the non-priority phase under strategy a is shown in Fig. 6. The shaded area is the decreased delay in the non-priority phase after green extension implementation. Equation (4) is the decreased delay of the private vehicles in non-priority phase due to green extension implemented in non-priority phase under strategy a.

4 $$\Delta d_{npD}^{a} = \frac{{t_{gnp}^{in} q_{np} \left[ {2t_{rnp} + t_{gnp}^{in} \left( {{{1 + q_{np} } \mathord{\left/ {\vphantom {{1 + q_{np} } {S_{np} }}} \right. \kern-0pt} {S_{np} }}} \right)} \right]}}{2}$$

where $\Delta d_{npD}^{a}$-Decreased delay of the non-priority phase due to the green extension in the non-priority phase under strategy a.

The increased delay of the priority phase due to the green extension in the non-priority phase under strategy a is shown in Fig. 7. The shaded area is the increased delay in the priority phase after green extension implementation. Equation (5) is the increased delay of the private vehicles in priority phase due to green extension implemented in non-priority phase under strategy a.

5 $$\Delta d_{pI}^{a} = \frac{{t_{gnp}^{in} q_{p} \left( {2t_{gp} + t_{gnp}^{in} - 2t_{gp}^{in} } \right)}}{{2\left( {1 - {{q_{p} } \mathord{\left/ {\vphantom {{q_{p} } {S_{p} }}} \right. \kern-0pt} {S_{p} }}} \right)}}$$

where $\Delta d_{pI}^{a}$-Increased delay of the priority phase due to the green extension in the non-priority phase under strategy a.

Equation (6) calculates the total delay reduction for private vehicles under strategy a, which is equal to the sum of Eq. (2) through Eq. (5).

6 $$\overline{d}_{a} = {{\left( {\Delta d_{pD}^{a} - \Delta d_{pI}^{a} } \right)} \mathord{\left/ {\vphantom {{\left( {\Delta d_{pD}^{a} - \Delta d_{pI}^{a} } \right)} {q_{p} C_{d}^{a} }}} \right. \kern-0pt} {q_{p} C_{d}^{a} }} + ({{\Delta d_{npD}^{a} - \Delta d_{npI}^{a} )} \mathord{\left/ {\vphantom {{\Delta d_{npD}^{a} - \Delta d_{npI}^{a} )} {q_{np} C_{d}^{a} }}} \right. \kern-0pt} {q_{np} C_{d}^{a} }}$$

where $C_{d}^{a}$-The length of the dynamic cycle under strategy a.

**Figure 8:**
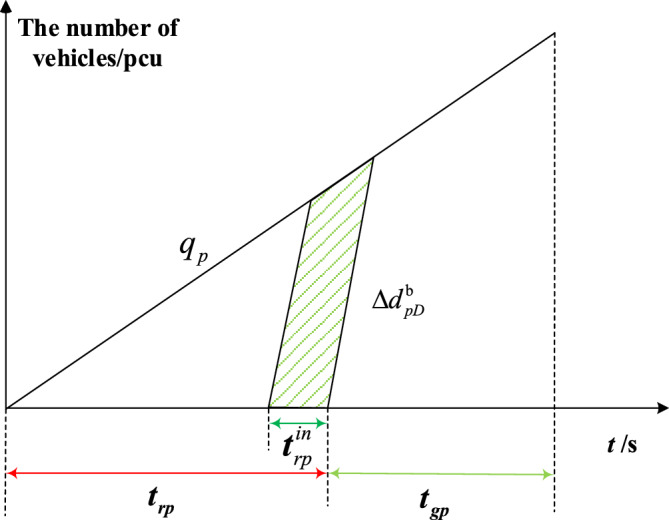
Decreased delay of the priority phase due to the red truncation in the priority phase under strategy b.

**Figure 9:**
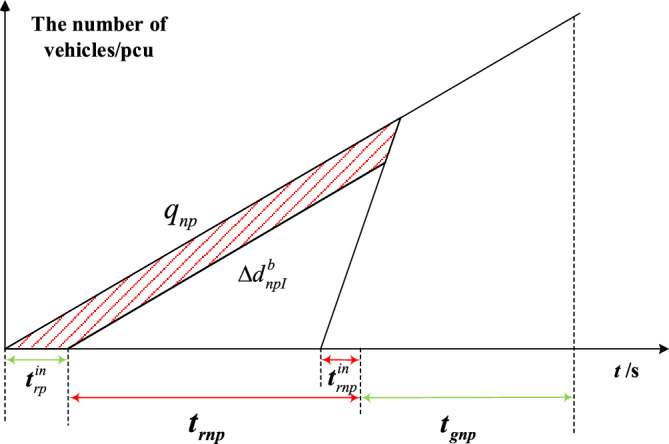
Increased delay of the non-priority phase due to the red truncation in the priority phase under strategy b.

**Figure 10:**
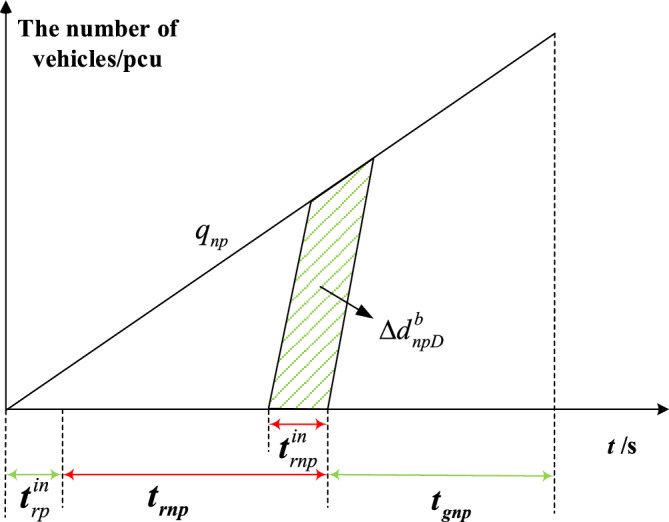
Decreased delay of the non-priority phase due to the red truncation in the non-priority phase under strategy b.

**Figure 11:**
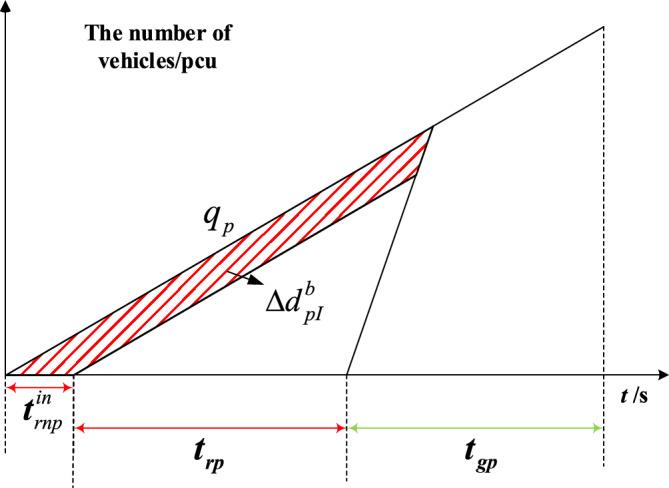
Increased delay of the priority phase due to the red truncation in the non-priority phase under strategy b.

Figures 8, 9, 10 and 11 represent the delay reduction of private vehicles between decreased delay and increased delay when the self-driving bus cannot pass through the intersection and the traffic light is in the red phase. Figures 8 and 9 show the delay of private vehicles in the priority phase and non-priority phase due to the red truncation in the priority phase. Figures 10 and 11 show the delay of private vehicles in the priority phase and non-priority phase due to the red truncation in the non-priority phase.

Figure 8 shows the decreased delay of the priority phase due to red truncation in the priority phase under strategy b. The shaded area is the decreased delay in the priority phase after red truncation implementation. Equation (7) calculates the decreased delay of the private vehicles in priority phase due to red truncation implemented in priority phase under strategy b.

7 $$\Delta d_{pD}^{b} = \frac{{q_{p} t_{rp}^{in} \left( {2t_{rp} - t_{rp}^{in} } \right)}}{{2\left( {1 - {{q_{p} } \mathord{\left/ {\vphantom {{q_{p} } {S_{p} }}} \right. \kern-0pt} {S_{p} }}} \right)}}$$

where $\Delta d_{pD}^{b}$-Decreased delay of the priority phase due to the red truncation in the priority phase under strategy b; $t_{rp}^{in}$-Red truncation time in the priority phase to ensure that the self-driving bus passes through the intersection without stopping.

Figure 9 shows the increased delay of the non-priority phase due to the red truncation in the priority phase under strategy b. The shaded area is the increased delay in the non-priority phase after red truncation implementation. Equation (8) calculates the increased delay of the private vehicles in non-priority phase due to red truncation implemented in priority phase under strategy b.

8 $$\Delta d_{npI}^{b} = \frac{{q_{np} t_{rp}^{in} \left( {t_{rp}^{in} - 2t_{rnp}^{in} + t_{rnp} } \right)}}{{2\left( {1 - {{q_{np} } \mathord{\left/ {\vphantom {{q_{np} } {S_{np} }}} \right. \kern-0pt} {S_{np} }}} \right)}}$$

where $\Delta d_{{npI}}^{b}$-Increased delay of the non-priority phase due to the red truncation in the priority phase under strategy b; $t_{{rnp}}^{{in}}$-Red truncation time in the non-priority phase for mitigating the increased delay in the non-priority phase.

The decreased delay of the non-priority phase due to the red truncation in the non-priority phase under strategy b is shown in Fig. 10. The shaded area is the decreased delay in the non-priority phase after red truncation implementation. Equation (9) is the decreased delay of the private vehicles in non-priority phase due to red truncation implemented in non-priority phase under strategy b.

9 $$\Delta d_{npD}^{b} = \frac{{q_{np} t_{rnp}^{in} \left( {2t_{rnp} + 2t_{rp}^{in} - t_{rnp}^{in} } \right)}}{{2\left( {1 - {{q_{np} } \mathord{\left/ {\vphantom {{q_{np} } {S_{np} }}} \right. \kern-0pt} {S_{np} }}} \right)}}$$

where $\Delta d_{npD}^{b}$-Decreased delay of the non-priority phase due to the red truncation in the non-priority phase under strategy b.

The increased delay of the priority phase due to red truncation in the non-priority phase under strategy b is shown in Fig. 11. The shaded area is the increased delay in the priority phase after red truncation implementation. Equation (10) is the increased delay of the private vehicles in priority phase due to red truncation implemented in non-priority phase under strategy b.

10 $$\Delta d_{pI}^{b} = \frac{{t_{rnp}^{in} q_{p} \left( {2t_{rp} + t_{rnp}^{in} } \right)}}{{2\left( {1 - {{q_{p} } \mathord{\left/ {\vphantom {{q_{p} } {S_{p} }}} \right. \kern-0pt} {S_{p} }}} \right)}}$$

where $\Delta d_{pI}^{b}$-Increased delay of the priority phase due to the red truncation in the non-priority phase under strategy b.

Equation (11) calculates the total delay reduction for private vehicles under strategy b, which is equal to the sum of Eq. (7) through Eq. (10).

11 $$\overline{d}_{b} = {{\left( {\Delta d_{pD}^{b} - \Delta d_{pI}^{b} } \right)} \mathord{\left/ {\vphantom {{\left( {\Delta d_{pD}^{b} - \Delta d_{pI}^{b} } \right)} {q_{p} C_{d}^{b} }}} \right. \kern-0pt} {q_{p} C_{d}^{b} }} + {{\left( {\Delta d_{npD}^{b} - \Delta d_{npI}^{b} } \right)} \mathord{\left/ {\vphantom {{\left( {\Delta d_{npD}^{b} - \Delta d_{npI}^{b} } \right)} {q_{np} C_{d}^{b} }}} \right. \kern-0pt} {q_{np} C_{d}^{b} }}$$

where $C_{d}^{b}$-The length of the dynamic cycle under strategy b.

### Lower model

**Figure 12:**
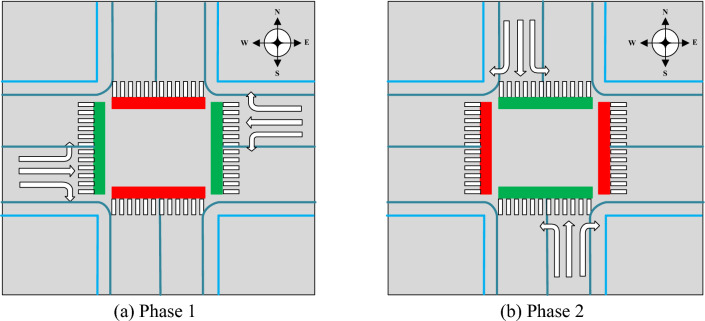
Phase diagram of the two-phase intersection.

Game theory is used to simulate the green time allocation, which can be regarded as a dynamic game of complete information^25^. The lower model is constructed according to the Stackelberg model. Taking the two-phase intersection as an example, the phase diagram is shown in Fig. 12. Phase 1 is the priority phase, and phase 2 is the non-priority phase.

The length of dynamic cycle $C_{d}$ equals the sum of the green time in each phase.

12 $$C_{d} = t_{gp}^{d} + t_{gnp}^{d}$$

where $t_{gp}^{d}$-Green time in phase 1; $t_{gnp}^{d}$-Green time in phase 2.

In the Stackelberg model, The payment functions $f_{1}$ and $f_{2}$ are calculated using Eqs. (13) and (14) to subtract the number of vehicles that have dissipated during the green time from the number of existing and arriving vehicles on the current lane.

13 $$f_{1} = \omega_{p} - S_{p} t_{gp}^{d} + q_{p} t_{gnp}^{d}$$

14 $$f_{2} = \omega_{np} - S_{np} t_{gnp}^{d} + q_{np} t_{gp}^{d}$$

where $\omega_{p}$-The number of vehicles waiting at phase 1; $\omega_{np}$-The number of vehicles waiting at phase 2.

In the Stackelberg model, the priority phase is treated to make decisions first, and the non-priority phase makes its own decision based on the observations from the priority phase. The green time in each phase under TSP is calculated according to the Stackelberg model. Equation (15) calculate the maximum value of dissipating vehicles on the road during the current time period, which is the maximum benefit function. The optimal response $t_{gnp}^{s}$ calculation for phase 2 is based on game theory:

15 $$\mathop {\max }\limits_{{_{gnp}^{d} \ge 0}} f_{2} (t_{gp}^{d} ,t_{gnp}^{d} ) = \mathop {\max }\limits_{{t_{gnp}^{d} \ge 0}} \left\{ {\left( {t_{2} - t_{gnp}^{d} } \right)\left( {\omega_{np} - S_{np} t_{gnp}^{d} + q_{np} t_{gp}^{d} } \right)} \right\}$$

where $t_{2}$-The green time in initial phase 2.

Equation (16) is based on the Stackelberg model, dominated by the decision made in the first stage, and the second stage calculates its optimal response red time based on the decision made in the first stage to shorten the green time.

16 $$t_{gnp}^{s} = R_{2} \left( {t_{gp}^{d} } \right) = \frac{{q_{np} \left( {\omega_{p} + S_{p} t_{1} } \right) + 2S_{p} \left( {\omega_{np} + S_{np} t_{2} } \right)}}{{4S_{p} S_{np} - q_{p} q_{np} }}$$

The optimal response function $t_{gp}^{s}$ of phase 1 can be predicted based on $R_{2} \left( {t_{gp}^{d} } \right)$ through complete information dynamic game calculation. Equation (17) calculates the maximum value of dissipating vehicles on the road during the current time period as the benefit function based on the uniqueness of the leader.

17 $$\mathop {\max }\limits_{{t_{gp}^{d} \ge 0}} f_{1} (t_{gp}^{d} ,R_{2} \left( {t_{gp}^{d} } \right)) = \mathop {\max }\limits_{{t_{gp}^{d} \ge 0}} \left\{ {\left( {t_{1} - t_{gp}^{d} } \right)\left( {\omega_{p} - S_{p} t_{gp}^{d} + q_{p} R_{2} \left( {t_{gp}^{d} } \right)} \right)} \right\}$$

where $t_{1}$-The green time in initial phase 1.

Equation (18) comprehensively considers the traffic status information of two phases and uses the Stackelberg model to calculate the green time in priority phase.

18 $$t_{gp}^{s} = \frac{{\omega_{p} }}{{2S_{p} }} + \frac{{t_{1} }}{2} + \frac{{q_{np} }}{{2S_{p} }}\frac{{\left( {\omega_{p} + S_{p} t_{1} } \right) + 2S_{p} \left( {\omega_{np} + S_{np} t_{2} } \right)}}{{4S_{p} S_{np} - q_{p} q_{np} }}$$

The factor determining the time length of green in each phase of the dynamic cycle is the vehicle arrival rate in this phase.

In strategy a, the green extension time in priority and non-priority phase are as follows:

19 $$t_{gp}^{in} = t_{gp}^{s} - t_{gp}^{{}}$$

20 $$t_{gnp}^{in} = t_{gnp}^{s} - t_{gnp} + t_{gp}^{in}$$

In strategy b, the red truncation time in priority and non-priority phase are as follows:

21 $$t_{rp}^{in} = t_{gnp}^{{}} - t_{gnp}^{s}$$

22 $$t_{rnp}^{in} = t_{gp} - t_{gp}^{s} + t_{rp}^{in}$$

The arrival rate of vehicles at each phase should be less than the saturation flow rate to avoid oversaturation. The length of the dynamic cycle should be greater than or equal to the minimum cycle length $C_{\min }$. This ensures that vehicles arriving at the intersection within a cycle are released precisely, without any stranded vehicles or excessive green light time.

subject to

23 $$q_{p} \le S_{p}$$

24 $$q_{np} \le S_{np}$$

25 $$C_{d} \ge C_{\min }$$

26 $$C_{\min } = \frac{L}{1 - Y}{ = }\frac{L}{{1 - \sum\limits_{k = 1}^{K} {y_{k} } }}{ = }\frac{L}{{1 - \sum\limits_{k = 1}^{K} {\frac{{q_{k} }}{{S_{k} }}} }}$$

where $L$-Total lost time for all critical phases; $Y$-Sum of the flow (volume/saturation flow) ratios for all critical phases; $y_{k}$-Flow ratio in critical phase $k$; $K$-The number of critical phases; $q_{k}$-Volume in critical phase $k$; $S_{k}$-Saturation flow rate in critical phase $k$.

27 $$L = \sum\limits_{k = 1}^{K} {(l_{k} + I - A)}$$

where $l_{k}$-Start-up lost time in critical phase $k$; $I$-Inter green time, that is, the yellow change interval plus red clearance interval; $A$-Yellow time.

### Car-following model

For self-driving vehicles, the rear vehicle can obtain the traffic status of the front vehicle and the surrounding traffic condition by V2X. CACC technology can be implemented. The PATH laboratory proposed the CACC following model^26^ based on the verification of small-scale platooning experiments. Equations (28) and (29) together create a feedback control system for automatic following of autonomous vehicles, allowing the speed of the following vehicle to be adjusted based on the state of the preceding vehicle, ensuring a safe distance is maintained. The equations are as follows:

28 $$v_{s} = v_{s - 1} + r_{p} \cdot e_{s - 1} + r_{d} \cdot e_{s - 1}$$

29 $$e_{s} = l_{{\text{c}}} - l_{m} - l_{\Delta } - \tau_{c} \cdot v_{s - 1}$$

where $v_{s}$-Speed of the subject vehicle at time step $s$; $v_{{s{ - }1}}$-Speed of the subject vehicle in the previous time step; $e_{s}$-Gap error of the subject vehicle at time step $s$; $e_{{s{ - }1}}$-Gap error of the subject vehicle in the previous time step; $r_{p}$-Gain is 0.45 s^−1^; $r_{d}$-Gain is 0.25; $l_{c}$-Intervehicle spacing; $l_{m}$-Spacing margin; $l_{\Delta }$-Vehicle length; $\tau_{c}$-Desired time gap of CACC.

## Solution

### Genetic algorithm

The genetic algorithm (GA) is used to solve the bi-level programming delay model. The coding length $L$ is 24. Individuals are encoded as binary strings of length DNA_SIZE*2. The population size $M$ is 200. The number of generations $N$ is 50. The maximum number of iterations $G$ is 100. The Fitness function is $F\left( {x,y} \right)$, which represents the delay reduction of private vehicles for a given vehicle arrival rate $x,y$. Depending on the individuals’ fitness values, the individuals are selected, crossed and mutated within the population. One of the individuals was used as the parent, and the first generation of the first offspring acquired all genes of the father. One individual is randomly selected as the mother, and the offspring acquires the mother's genes at the crossover position (randomly generated, which can be any position on the chromosome). The mutation probability of the offspring is 0.003. The values of the crossover probability and mutation probability determine the local and global optimal solution searchability of the algorithm. The mutation probability of offspring is 0.003. $P_{{\text{c}}}$ takes 0.7. $P_{{\text{m}}}$ takes 0.005. The crossover and mutation individuals are added to the new population, and each genotype is iterative. An array is transformed and selected to generate a new population.

The pseudocode of the GA is illustrated in Table 1. The functions are encoded as binary functions. Individual $q_{p,np}$ is represented by each element of the array. The objective function is the fitness function.

**Table 1:**
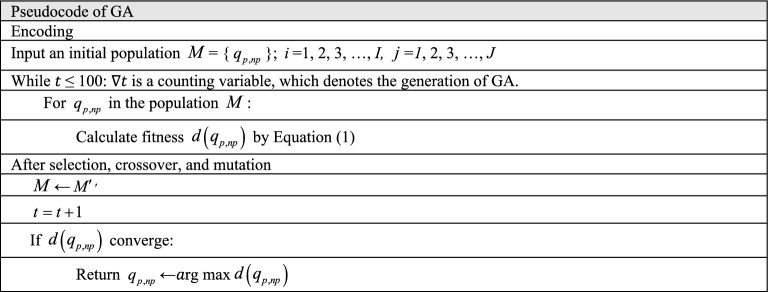
Pseudocode of GA.

## Case study

### Case description

**Figure 13:**
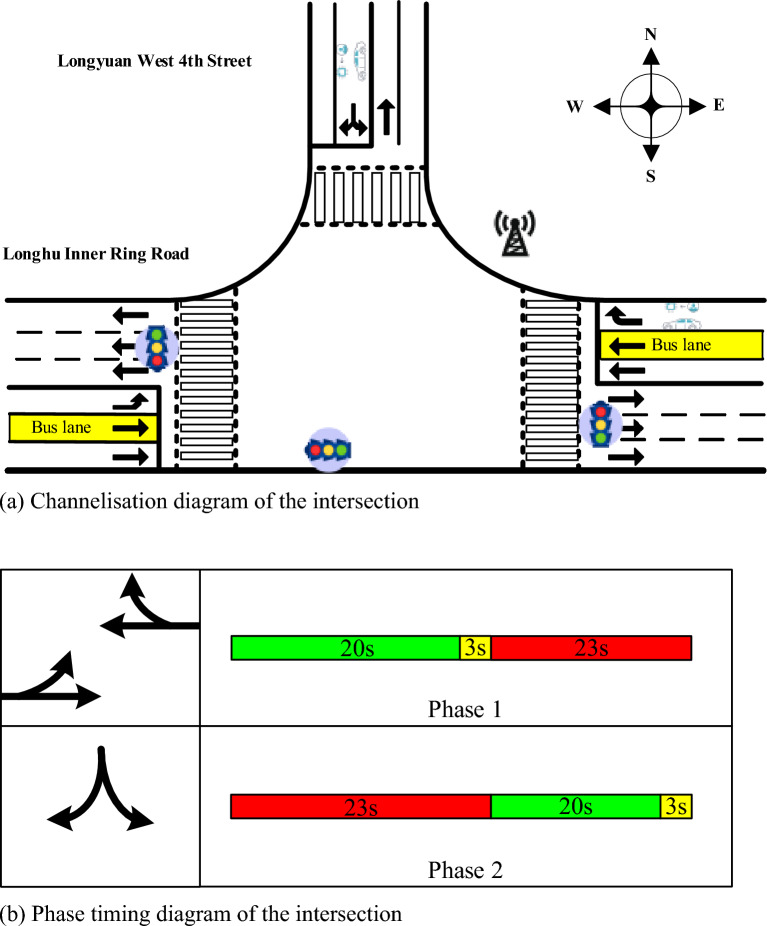
Intersection information of the Longhu inner ring road and Longyuan west 4th street.

A self-driving bus in the city of Zhengzhou is used in this section for the case study. Test runs of self-driving buses were held in 2019 by combining technologies, such as 5G network services and artificial intelligence with smart supervision and control systems. Self-driving buses are connected with traffic signal lights and other vehicles by RSUs and traffic data are obtained by OBUs. At present, the self-driving bus line passes through 29 signalised intersections. Four-phase signalised intersections account for 24.14%, three-phase signalised intersections account for 13.79%, and two-phase signalised intersections account for 62.07%. Therefore, a two-phase signalised intersection was chosen to study the impact of the self-driving bus signal priority on the vehicle, as shown in Fig. 13.

### Genetic algorithm

**Figure 14:**
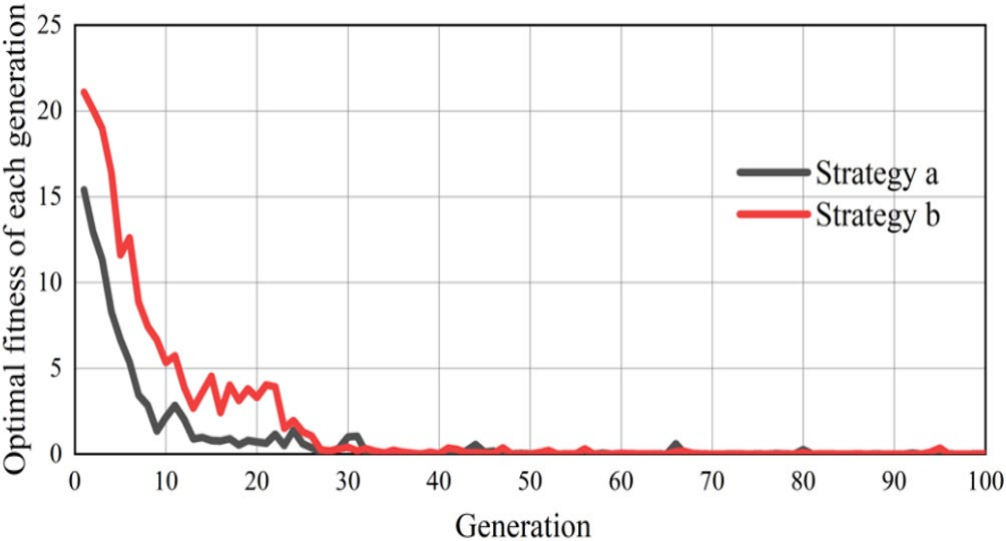
Convergence process of GA.

Python 3.8 is used to solve the bi-level programming delay model. When the length of the dynamic cycle is less than $C_{\min }$, the delay is calculated by $C_{\min }$. The convergence process of the GA is shown in Fig. 14. It can be observed that strategy a and strategy b converged when the curve was close to the 30th generation. The average convergence time is recorded as 3.37 s, as shown in Table 2. It is worth noting that the model's calculations take place only when the self-driving bus enters the designated detection zone, which is located 70 m away from the intersection. The length of detection zone is determined according to the stopping sight distance in “Code for design of urban road engineering (CJJ 37-2016)”^27^. Considering the self-driving bus operates at an average speed of 25 km/h, it takes approximately 10 s for the bus to traverse the distance from the starting point of the detection zone to the intersection. Therefore, with this computation time, the model demonstrates sufficient capability for real-time applications.

**Table 2:** Convergence time.

1	2	3	4	5	6	7	8	9	10	Mean
3.14	3.22	3.67	3.38	3.37	3.4	3.37	3.36	3.41	3.40	3.37

**Figure 15:**
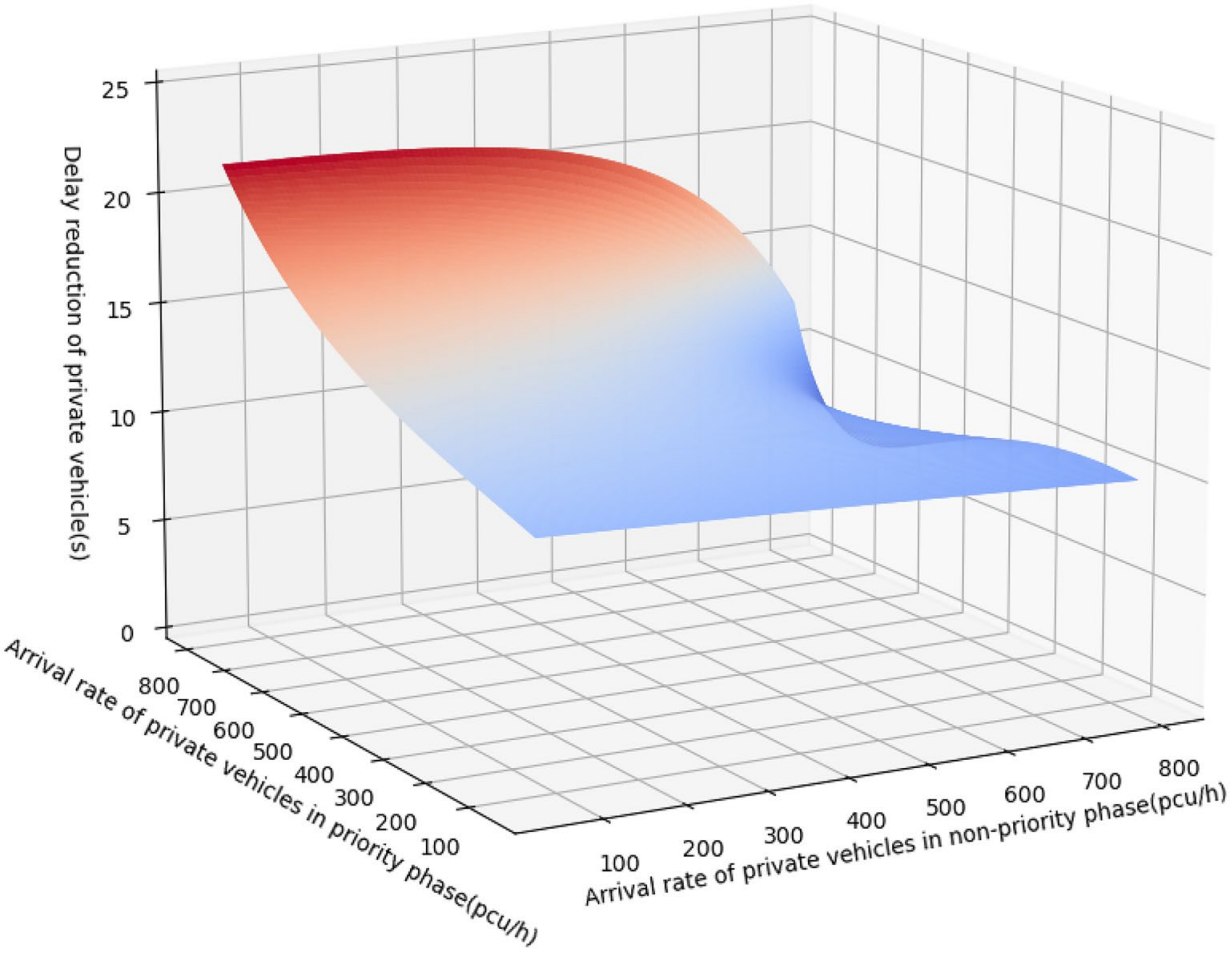
Delay reduction in the experimental period under different arrival rates in the priority and non-priority phases.

Figure 15 shows the delay reduction in the experimental period under different arrival rates in the priority and non-priority phases by GA.

It can be seen in Fig. 15 that with the increasing arrival rate of private vehicles, the delay reduction grows. The change in the delay reduction is not noticeable when the volume of the intersection is small or the arrival rate of private vehicles in the non-priority phase is much greater than the arrival rate in the priority phase. With the complex road environment and higher arrival rate, the proposed method guarantees the self-driving bus passing intersection without stopping and reduces the delay of private vehicles due to the implementation of TSP.

### Simulation

SUMO is used to simulate the case by the CACC car-following model. The traffic control interface (TraCI) of SUMO is programmed in Python, and then the SUMO-Python simulation experiment environment is constructed.

The speed of the self-driving bus is 20–25 ${\text{km}}/{\text{h}}$ in the link, and it is 15–20 ${\text{km}}/{\text{h}}$ at the intersection. Due to the randomness of the simulation, the model is performed 10 times, and the results are averaged. A dynamic cycle is carried out when the self-driving bus passes through the intersection, and the original signal phase is used after the self-driving bus passes through the intersection. The simulation time is 3600 s. Parameters of the car-following model from SUMO are as follows. The speedControlGainCACC is -0.4. The gapClosingControlGainGap is 0.005. The gapClosingControlGainGapDot is 0.05. The gapControlGainGap is 0.45. The gapControlGainGapDot is 0.0125. The collisionAvoidanceGainGap is 0.45. The collisionAvoidanceGainGapDot is 0.05. According to the interpretation of driving behavior parameters (Cautious, Normal, Aggressive) by Lyu et al.^28^, the self-driving behavior in this paper is cautious, based on the following acceleration condition (FAC), the following deceleration condition (FDC), the free cruise condition (FCC), the following steady condition (FSC), the relatively distant condition (RDC), the relative approximation condition (RAC) and the lane changing (LC).

**Figure 16:**
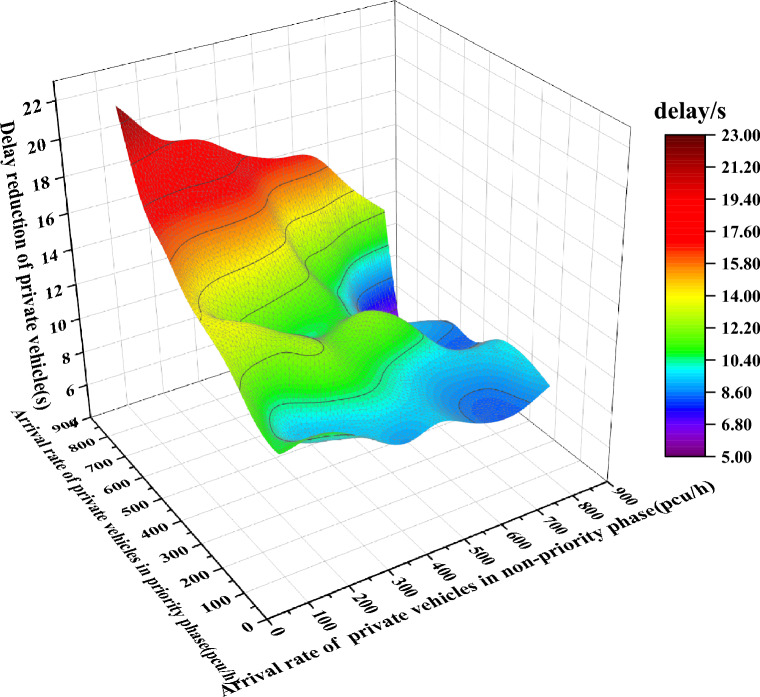
Delay reduction between scenario 3 and scenario 1.

There are three scenarios. Scenario 1 is where the transit signal priority is not implemented, and the self-driving bus has no priority. Scenario 2 is active signal priority, that is, the self-driving bus has priority, but the delay in the non-priority phase is not considered. Scenario 3 is dynamic signal priority, that is, the self-driving bus has priority, and the delay in the non-priority phase is considered. The delay reduction between Scenario 3 and Scenario 1 is shown in Fig. 16.

Comparing Figs. 15 and 16, the trend of the delay reduction is the same. With the increasing arrival rate of private vehicles, the delay reduction grows. The change in the delay reduction is not noticeable when the volume of the intersection is small or the arrival rate of private vehicles in the non-priority phase is much greater than the arrival rate in the priority phase.

A comparison of the delay reduction of private vehicles from SUMO and GA is shown in Table 3.

**Table 3:** Comparison of the delay reduction of private vehicles from SUMO and GA (the values in parentheses are from GA).

Non-priority phase	100	200	300	400	500	600	700	800
Priority phase								
100	10.58 (12.80)	11.34 (11.52)	10.32 (10.34)	8.75 (10.12)	9.56 (10.08)	8.54 (9.97)	8.25 (8.58)	9.21 (8.46)
200	11.28 (12.94)	9.45 (10.74)	9.28 (10.35)	10.11 (10.21)	9.58 (10.04)	8.72 (9.66)	8.54 (9.31)	9.85 (9.23)
300	12.89 (13.63)	10.58 (11.55)	12.32 (11.09)	11.21 (10.81)	10.34 (10.66)	9.57 (9.81)	9.58 (9.24)	9.68 (9.77)
400	13.56 (14.86)	13.65 (12.64)	12.42 (11.83)	11.45 (11.53)	12.26 (11.77)	11.24 (11.50)	8.51 (9.17)	8.38 (9.90)
500	14.57 (14.97)	12.41 (13.12)	11.23 (12.26)	10.45 (8.55)	10.85 (10.65)	9.45 (9.12)	9.28 (8.28)	8.57 (7.63)
600	16.34 (16.12)	14.57 (15.20)	13.97 (14.70)	12.81 (13.23)	11.56 (12.73)	9.38 (9.85)	7.45 (7.62)	7.19 (6.52)
700	17.58 (18.50)	16.97 (17.40)	16.89 (17.37)	16.16 (15.40)	12.46 (13.78)	11.18 (9.56)	6.45 (7.46)	4.31 (4.67)
800	22.45 (21.79)	19.65 (19.79)	19.21 (19.49)	17.67 (16.58)	17.09 (16.01)	16.78 (15.54)	14.51 (12.97)	12.92 (11.78)

It can be seen in Table 3 that the error in the delay reduction from SUMO and GA is 6.73% on average, and the results are basically consistent. This proves the effectiveness of the proposed model.

The change in the delay of private vehicles between scenario 3 and scenario 1 is shown in Table 4. Table 5 illustrates the change in the delay of private vehicles between scenario 3 and scenario 2. The first row of the table is the arrival rate of private vehicles in the non-priority phase, and the first column of the table is the arrival rate of private vehicles in the priority phase.

**Table 4:** The change in the delay of private vehicles under different arrival rates between scenario 3 and scenario 1.

Non-priority phase	100	200	300	400	500	600	700	800
Priority phase								
100	26.12%	31.45%	26.94%	23.87%	23.60%	22.43%	22.97%	24.36%
200	22.10%	17.45%	17.78%	19.68%	18.67%	17.25%	17.43%	19.41%
300	23.93%	20.09%	22.90%	20.27%	19.87%	18.16%	18.18%	17.62%
400	26.79%	25.54%	21.77%	19.86%	21.19%	20.04%	14.45%	14.98%
500	24.29%	20.74%	18.77%	16.66%	18.14%	16.44%	16.06%	14.09%
600	26.48%	23.85%	23.62%	22.10%	20.12%	15.49%	12.32%	12.08%
700	29.72%	28.13%	26.51%	26.75%	20.44%	17.95%	10.20%	6.93%
800	33.38%	29.77%	29.64%	28.07%	28.75%	26.37%	22.10%	21.30%

**Table 5:** The change in the delay of private vehicles under different arrival rates between scenario 3 and scenario 2.

Non-priority phase	100	200	300	400	500	600	700	800
Priority phase								
100	20.47%	38.17%	23.95%	23.81%	13.14%	21.84%	22.36%	21.22%
200	21.14%	12.24%	15.92%	18.89%	23.79%	15.19%	22.77%	22.68%
300	30.83%	32.18%	28.57%	22.60%	30.83%	23.79%	23.80%	24.84%
400	34.06%	29.94%	25.63%	19.69%	21.57%	22.13%	12.60%	17.53%
500	25.77%	21.60%	19.54%	15.47%	19.48%	16.07%	17.80%	12.14%
600	27.45%	26.50%	27.98%	25.25%	24.26%	16.13%	16.05%	16.07%
700	33.13%	27.51%	21.68%	25.98%	21.18%	13.49%	7.62%	9.33%
800	31.97%	31.46%	31.74%	27.25%	32.36%	30.25%	16.65%	21.16%

The delay of private vehicles with dynamic signal priority (scenario 3) declined by 21.32% on average compared to that without priority (scenario 1). The delay of private vehicles with dynamic signal priority (scenario 3) declined by 22.63% on average compared with that with active signal priority (scenario 2). The proposed method is superior to the active signal priority.

The proposed method is compared with the method proposed by Yang et al.^29^. The results are shown in Table 6. It can be seen in Table 6 that the delay per private vehicle is 14.43 s in this paper, and it is less than the delay from Yang et al. By comparing with the delay without TSP, in this paper the delay was reduced by 12.17%; however, the delay from Yang et al. increased by 11.11%. The effectiveness of the proposed method is further illustrated.

**Table 6:** Comparison of calculation results.

Methods	Delay per private vehicle with TSP(s)	Delay reduction compared without TSP
Proposed method in this paper	14.43	12.17%
Method proposed by Yang et al.	19.05	− 11.11%

## Conclusion

Transit signal priority (TSP) is an important means to improve the speed and reliability of the bus system. However, the TSP will lead to the delay of private vehicles, especially in the non-priority phase. How to balance the interests of buses and private vehicles is an urgent problem to be solved. With the development of self-driving technology, it can be effectively alleviated. Therefore, this paper discusses the delay reduction of private vehicles after the implementation of self-driving bus signal priority. A bi-level programming model is proposed to balance the benefits of self-driving buses and private vehicles from the perspective of private vehicles, based on the arrival rate of private vehicles in the priority and non-priority phases. The genetic algorithm (GA) is used to calculate the model.

Based on the Self-driving Bus in Zhengzhou, a case study is illustrated to verify the effectiveness of the proposed model. The results from SUMO are compared with the calculation results by GA. It is found that the results are basically consistent, which verifies the effectiveness of the proposed model. By comparing with the results that without priority and active signal priority, the delay of private vehicles with dynamic signal priority is less. Then, the proposed method is compared to other papers, the delay per private vehicle is less than that of other papers, and the effectiveness of the proposed method is further illustrated. It can effectively improve the operation efficiency of the intersections and provide a reference for the implementation of self-driving bus signal priority.

However, there are still some limitations in this study. The research object in this paper is the isolated signalised intersection. TSP is implemented on bus lines, and it is set up on multiple roads. Therefore, coordinated transit signal priority is necessary in multiple signalised intersections. We will study the delay of private vehicles in arterial coordinated road conditions and achieve dynamic signal priority in complex traffic scenarios and continuous signal light requests. Self-driving buses and private vehicles are all under a connected environment in this paper; however, a mixed traffic environment with human-driven vehicles and connected and autonomous vehicles will be more common in the future. The penetration rates of self-driving buses and private vehicles will be considered in future work. The proposed method is used at the two-phase signalized intersection. In order to verify the generalization applicability of the proposed method, it will be implemented at the four-phase or even more-phase signalized intersections in future extensions.

## Data Availability

The data that support the findings of this study are available from the corresponding author, [Shuxin Li], upon reasonable request.
